# Supplementary material for: Highly-stable, injectable, conductive hydrogel for chronic neuromodulation
Source: Nat Commun. 2024 Sep 12;15:7993. doi: 10.1038/s41467-024-52418-y (PMC11393409; doi:10.1038/s41467-024-52418-y)
Supplement: Supplementary file 1 — Supplementary Information [file 41467_2024_52418_MOESM1_ESM.pdf]

Supplementary Information

# Highly-stable, injectable, conductive hydrogel for chronic neuromodulation


*Ming Yang[a#], Lufang Wang[b#], Wenliang Liu[a#], Wenlong Li[a], Yewei Huang[c], Qiaofeng Jin[b], Li Zhang[b*], Yuanwen Jiang[c*], and Zhiqiang Luo[a*]*

[a] National Engineering Research Center for Nanomedicine, College of Life Science and Technology, Huazhong University of Science and Technology, Wuhan, 430074, China.

[b] Department of Ultrasound Medicine, Union Hospital, Tongji Medical College, Huazhong University of Science and Technology, Wuhan, 430022, China.

[c] Department of Materials Science and Engineering, University of Pennsylvania, Philadelphia, 19104, US.

[#] These authors contributed equally: Ming Yang, Lufang Wang, and Wenliang Liu.

***Corresponding Authors**

*(Li Zhang) Email: zli429@hust.edu.cn

*(Yuanwen Jiang) Email: ywjiang@seas.upenn.edu

*(Zhiqiang Luo) Email: zhiqiangluo@hust.edu.cn

# Contents

**Supplementary Methods**

Supplementary Fig. 1 Structural characterization of 8-arm PEG-SH.

Supplementary Fig. 2 Structural characterization of the synthesized MXene.

Supplementary Fig. 3 Injectability of ICAA hydrogel on a random, rough, and deformable surface.

Supplementary Fig. 4 Influence of environmental variables on the gelation time of ICAA hydrogel.

Supplementary Fig. 5 Rheological properties of the ICAA hydrogel.

Supplementary Fig. 6 Compressive properties of the ICAA hydrogel.

Supplementary Fig. 7 Swelling properties of ICAA hydrogel.

Supplementary Fig. 8 Long-term stability of ICAA hydrogel.

Supplementary Fig. 9 Adhesion of the ICAA hydrogel on diverse tissues.

Supplementary Fig. 10 Janus adhesion of the ICAA hydrogel on diverse tissues.

Supplementary Fig. 11 Adhesion of the ICAA hydrogel with sciatic nerve tissues.

Supplementary Fig. 12 Adhesion of the ICAA hydrogel on PDA-coated substrates.

Supplementary Fig. 13 XPS analysis of MXene, MXene/PP and MXene/PP/TA.

Supplementary Fig. 14 AFM analysis of MXene, MXene/PP and MXene/PP/TA.

Supplementary Fig. 15 Conductivities of the ICAA hydrogel with different components.

Supplementary Fig. 16 CIC curves of the MH-interface with low stimulation voltage.

Supplementary Fig. 17 Electrochemical properties of the ICAA hydrogel under prolonged electrical stimulation.

Supplementary Fig. 18 Cytocompatibility of the ICAA hydrogel.

Supplementary Fig. 19 Injectable process of the ICAA hydrogel.

Supplementary Fig. 20 Histological analysis of vagus nerves at the device-implanted sites.

Supplementary Fig. 21 Monitoring of health status of rats during vagus nerve stimulation therapy.

Supplementary Fig. 22 Histological analysis of hearts.

Supplementary Fig. 23 Echocardiography imaging of rats in the control, MI, and ICAA-C groups 1 day and 2 weeks after modeling.

Supplementary Fig. 24 End-diastolic diameters (a) and volumes (b) of hearts in the ICAA-C, MI, and control groups within 4 weeks.

Supplementary Fig. 25 End-systolic diameters (a) and volumes (b) of hearts in the ICAA-C, MI, and control groups within 4 weeks.

Supplementary Fig. 26 Representative immunofluorescence images of the infarct area in the ICAA-C, MI, and control groups for wheat germ agglutinin (WGA).

Supplementary Fig. 27 Immunohistochemical analysis of collagen expression in the ICAA-C, MI, and control groups.

Supplementary Fig. 28 Histological analysis of different organs.

Supplementary Fig. 29 Effects of ICAA-C-enabled vagus nerve stimulation on neurotransmitters released by the sympathetic nerves.

Supplementary Fig. 30 Representative immunofluorescence images of GAP-43 and TH.

Supplementary Table 1 Component content of the ICAA hydrogels.

Supplementary Table 2 Assignments of Ti *2p* XPS spectra of MXene, MXene/PP and MXene/PP/TA.

Supplementary Table 3 Component content of the ICAA hydrogels with different conductive components and TA contents.

Supplementary Table 4 Comparison of the ICAA hydrogel with previously reported injectable, conductive hydrogels.

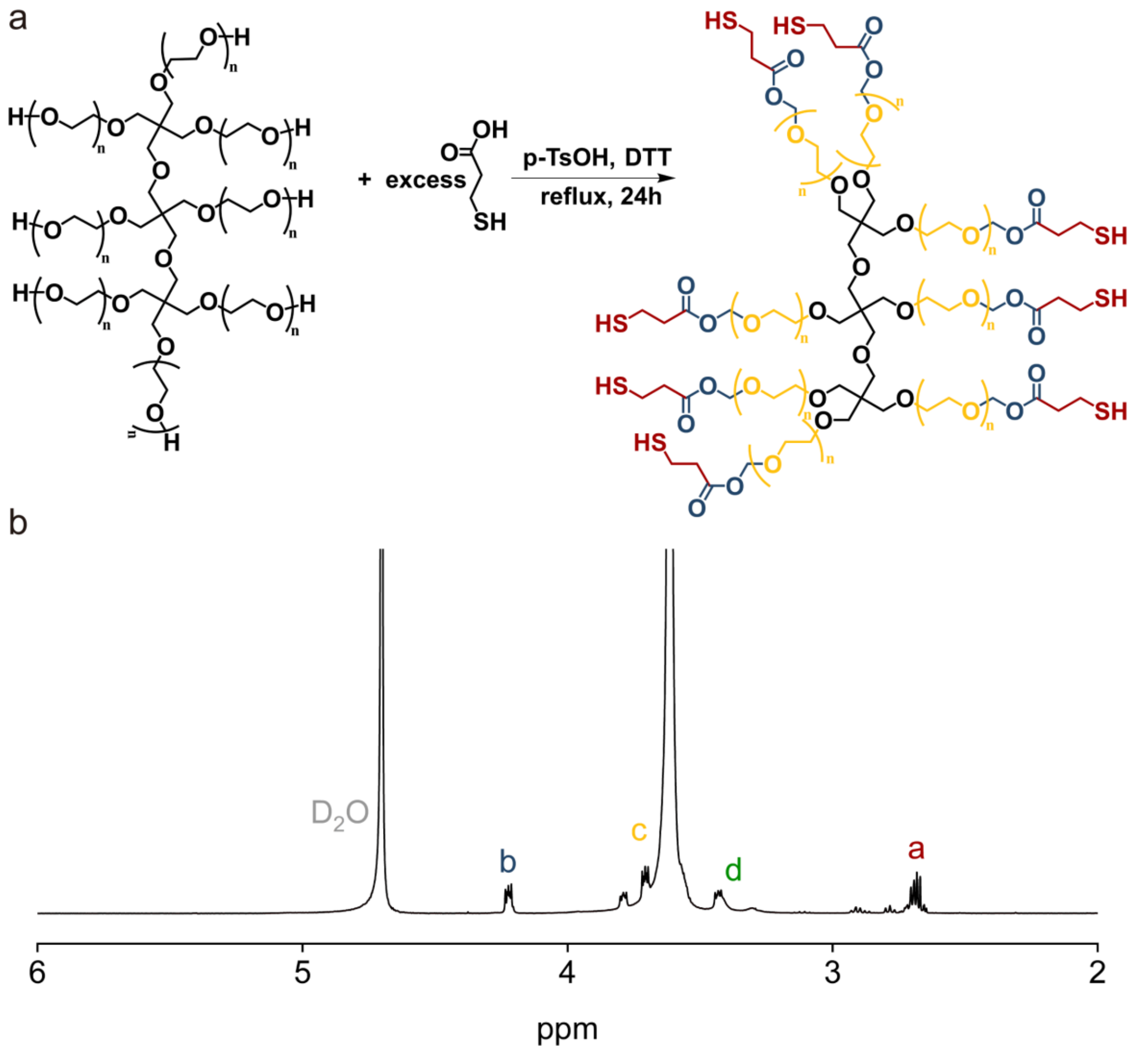


**Supplementary Fig. 1 Structural characterization of PEG-8SH. a** Schematic of the synthesis of 8-arm PEG-8SH (MW of 8-arm PEG = 20000, n = 55.7 repeating units). **b** $^{1}$H NMR spectrum of PEG-8SH (400 MHz, 25 °C, $D_2O$). The thiol functionality of the PEG-8SH as determined via 1H NMR spectroscopy was 7.09. PEG-8SH derivatives: 1H NMR ($D_2O$): δ=4.22 (16H, $-CH_2OC(O)-$, m), 3.79-3.6 (1782H, PEG backbone, $-CH_2CH_2O-$, s), 3.3-3.43 (24H, $-C-O-CH_2$) 2.9-2.68 (32H, $-CH_2CH_2SH$, m).

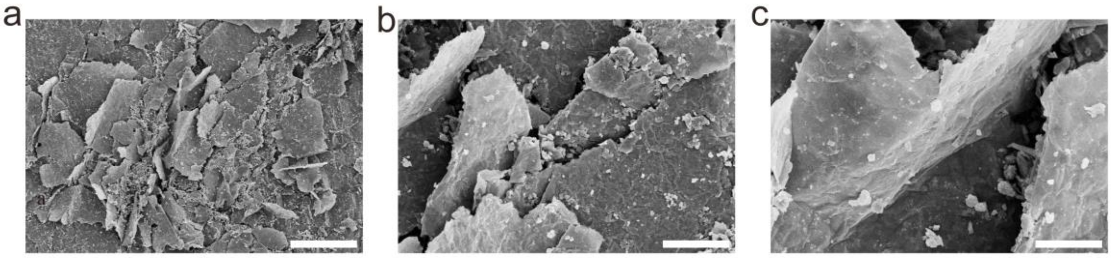


**Supplementary Fig. 2 Structural characterization of the synthesized MXene.** The (a-c) SEM images of MXene nanosheets in different magnification. Scale bars, 25 μm (a), 5 μm (b), 2.5 μm (c).

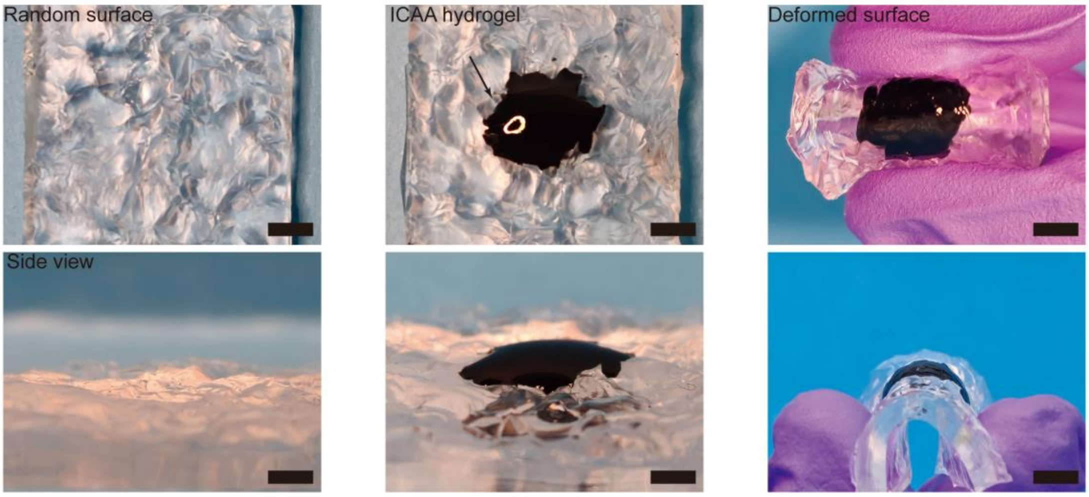


**Supplementary Fig. 3 Injectability of ICAA hydrogel on a random, rough, and deformable surface.** The ICAA precursors was injected on the tissue surface, and the ICAA hydrogel was formed *in-situ*, revealing its injectability and conformability. scale bar, 5 mm.

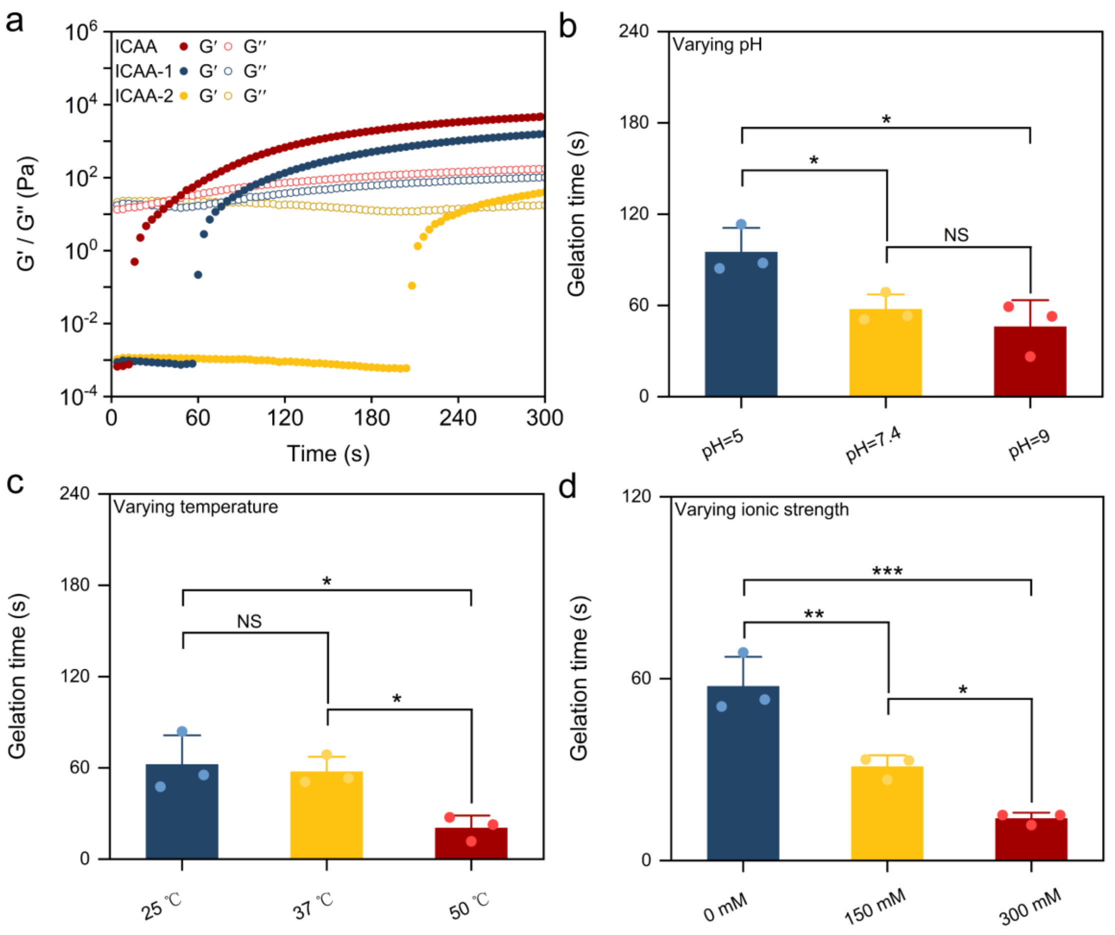


**Supplementary Fig. 4 Influence of environmental variables on the gelation time of ICAA hydrogel. a** Rheological G′ and G″ variation of ICAA hydrogel show adjustable gelation time by changing its solid contents (n = 3 independent experiments). At the gelation point, the storage modulus (G′) exceeds the loss modulus (G″). **b** Influence of pH on the gelation time of ICAA hydrogel (n = 3 independent experiments). **c** Influence of temperature on the gelation time of ICAA hydrogel (n = 3 independent experiments). **d** Influence of ionic strength on the gelation time of ICAA hydrogel (n = 3 independent experiments). The increase of pH, temperature or ionic strength shortens the gelation time of ICAA hydrogel. Data are presented as the mean ± standard deviation in (**b**), (**c**) and (**d**), and were analyzed by one-way ANOVA first, and then by the Tukey′s post hoc test. ***P ≤ 0.001. **b** $p$ = 0.0458 (pH 7.4 vs pH 5), $p$ = 0.01511 (pH 9 vs pH 5), $p$ = 0.63 (pH 9 vs pH 7.4). **c** $p$ = 0.9 (37 °C vs 25 °C), $p$ = 0.0195 (50 °C vs 25 °C), $p$ = 0.0325 (50 °C vs 37 °C). **d** $p$ = 0.00429 (150 mM vs 0 mM), $p = 3 \times 10^{-4}$ (300 mM vs 0 mM), $p$ = 0.032 (300 mM vs 150 mM).

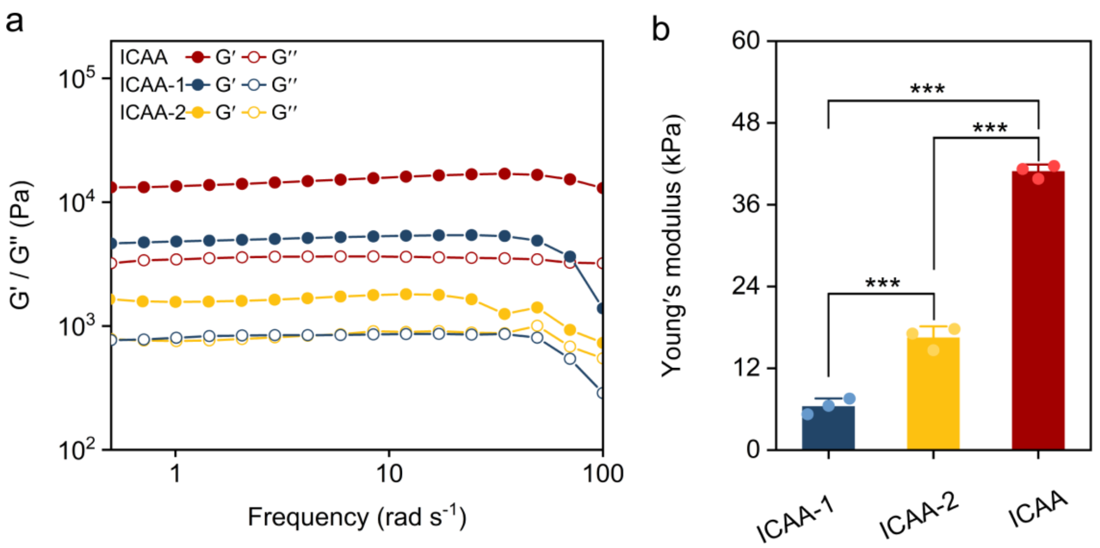


**Supplementary Fig. 5 Rheological properties of the ICAA hydrogel. a** Rheological frequency sweep tests over a frequency range from 0.5 to 100 rad·$s^{-1}$. **b** Young′s modulus of ICAA hydrogels (n = 3 independent experiments). Data are presented as the mean ± standard deviation in (**b**) and were analyzed by one-way ANOVA first, and then by the Tukey′s post hoc test. ***P ≤ 0.001. **b** $p = 1.85 \times 10^{-4}$ (ICAA-2 vs ICAA-1), $p = 8.49 \times 10^{-8}$ (ICAA vs ICAA-1), $p = 1.01 \times 10^{-6}$ (ICAA vs ICAA-2).

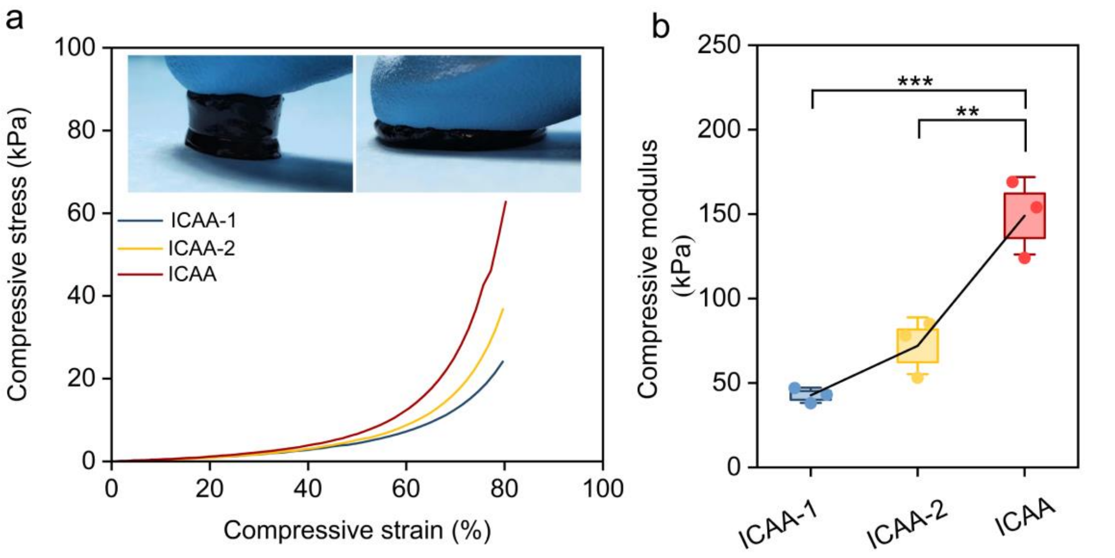


**Supplementary Fig. 6 Compressive properties of the ICAA hydrogel. a** Representative compressive stress-strain curves of the ICAA hydrogels show increased compressive modulus with the increase in the hydrogel's solid content. **b** Quantitative analysis of compressive modulus of the ICAA hydrogels reveals its tissue-like and adjustable compressive properties (n = 3 independent experiments). Data are presented as the mean ± standard deviation in (**b**) and were analyzed by one-way ANOVA first, and then by the Tukey′s post hoc test. $^{**}P \leq 0.01$. **b** $p = 5.58 \times 10^{-4}$ (ICAA vs ICAA-1), $p = 3.11 \times 10^{-3}$ (ICAA vs ICAA-2).

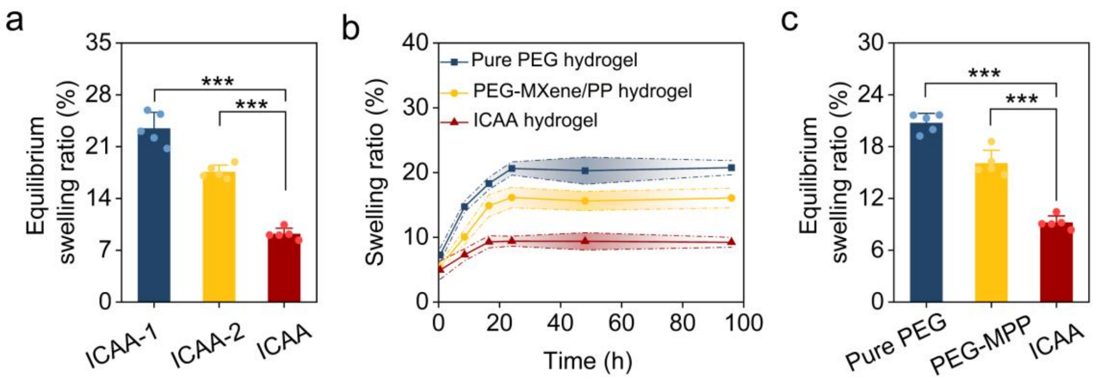


**Supplementary Fig. 7 Swelling properties of the ICAA hydrogel. a** Equilibrium swelling ratios of the ICAA hydrogels show significantly decrease when increasing their solid content (n = 5 independent experiments). **b** Swelling performance of ICAA, pure PEG, and PEG-MXene/PP hydrogel in PBS solution for 96 h (n = 5 independent experiments). **c** Equilibrium swelling ratios of ICAA, pure PEG, and PEG-MXene/PP hydrogel (n = 5 independent experiments). Data are presented as the mean ± standard deviation in (**a, b, c**) and were analyzed by one-way ANOVA first, and then by the Tukey′s post hoc test in (**a, c**). ***P ≤ 0.001. **a** $p = 0$ (ICAA vs ICAA-1), $p = 2.17 \times 10^{-6}$ (ICAA vs ICAA-2). **c** $p = 0$ (ICAA vs Pure PEG), $p = 2.05 \times 10^{-6}$ (ICAA vs PEG-MPP).

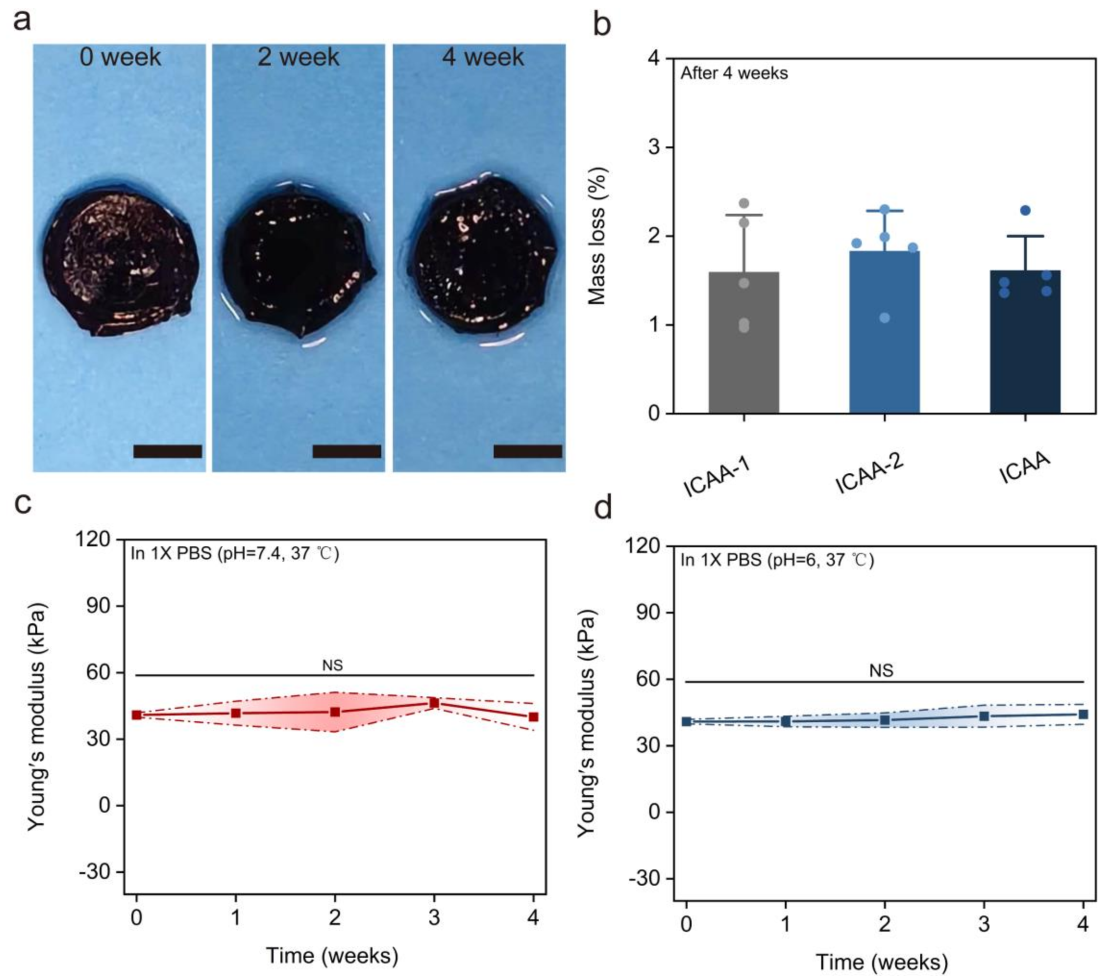


**Supplementary Fig. 8 Long-term stability of ICAA hydrogel. a** Photographs of the ICAA hydrogel immersed in 1× PBS buffer during 4 weeks. Scale bar, 5 mm. **b** Mass loss of the ICAA, pure PEG, PEG-MXene/PP hydrogels (n = 5 independent experiments). **c** Mechanical durability of ICAA hydrogel immersed in 1× PBS buffer (pH=7.4, 37 °C) during 4 weeks (n = 3 independent experiments). **d** Mechanical durability of ICAA hydrogel immersed in 1× PBS buffer (pH = 6, 37 °C) during 4 weeks (n = 3 independent experiments). Data are presented as the mean ± standard deviation in (**b, c, d**), and were analyzed by one-way ANOVA first, and then by the Tukey′s post hoc test. NS, not significant. **c** $p$ = 0.9997 (Week 1 vs Week 0), $p$ = 0.9979 (Week 2 vs Week 0), $p$ = 0.99995 (Week 2 vs Week 1), $p$ = 0.75141 (Week 3 vs Week 0), $p$ = 0.84194 (Week 3 vs Week 1), $p$ = 0.89051 (Week 3 vs Week 2), $p$ = 0.99966 (Week 4 vs Week 0), $p$ = 0.99521 (Week 4 vs Week 1), $p$ = 0.98658 (Week 4 vs Week 2), $p$ = 0.64689 (Week 4 vs Week 3). **d** $p$ = 0.96602 (Week 1 vs Week 0), $p$ = 0.99944 (Week 2 vs Week 0), $p$ = 0.99916 (Week 2 vs Week 1), $p$ = 0.90838 (Week 3 vs Week 0), $p$ = 0.91614 (Week 3 vs Week 1), $p$ = 0.96976 (Week 3 vs Week 2), $p$ = 0.78211 (Week 4 vs Week 0), $p$ = 0.79383 (Week 4 vs Week 1), $p$ = 0.8891 (Week 4 vs Week 2), $p$ = 0.99815 (Week 4 vs Week 3).

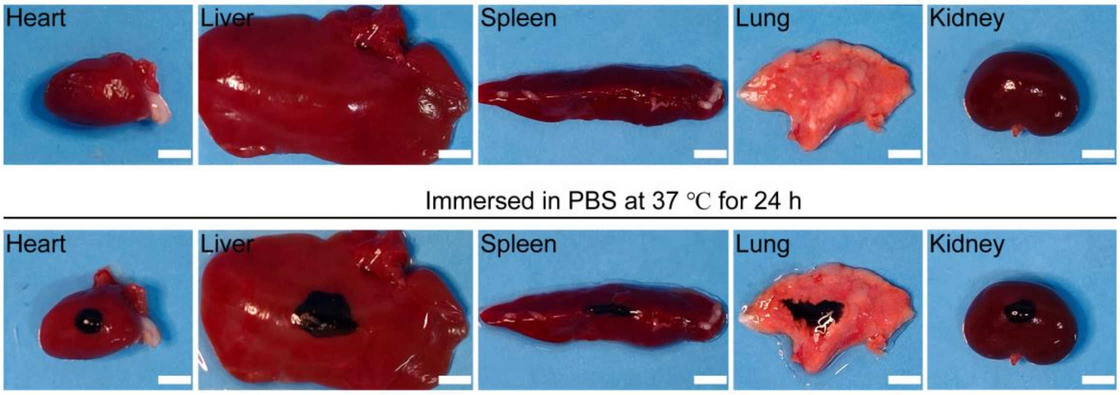


**Supplementary Fig. 9 Adhesion of the ICAA hydrogel on diverse tissues.** The ICAA precursors was injected on the tissue surface, and the ICAA hydrogel was formed *in-situ*, which maintained its adhesion after immersing in PBS at 37 ℃ for 24 h. Scale bars, 0.5 mm.

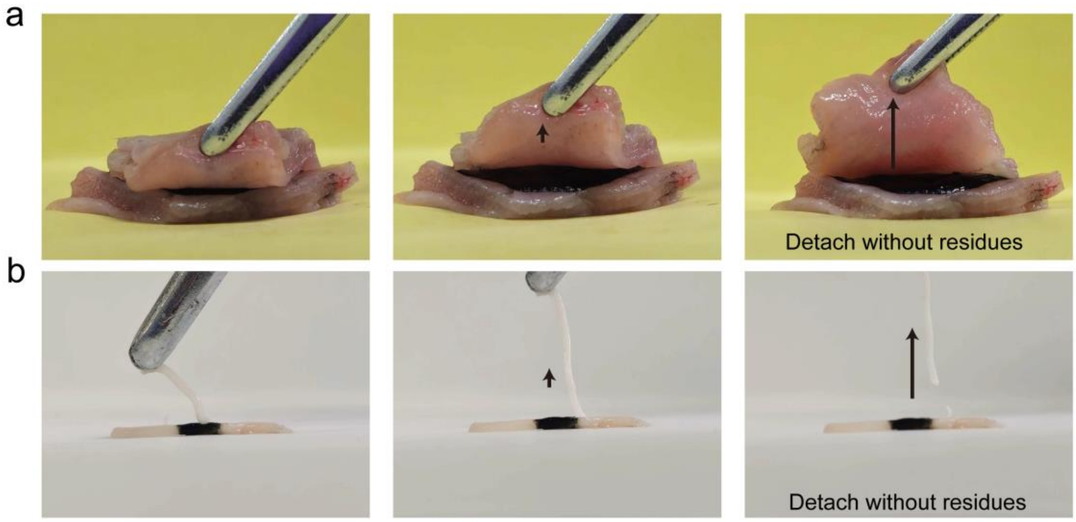


**Supplementary Fig. 10 Janus adhesion of the ICAA hydrogel on diverse tissues.** **a** Janus adhesion of the ICAA hydrogel with rat′s muscles. **b** Janus adhesion of the ICAA hydrogel with rat′s nerves. In the gel state, ICAA hydrogels possess dense cross-linking that restricts the diffusion of polymers and feature a smooth wet surface, avoiding unwanted adhesion to surrounding tissues and reduces the likelihood of post-operative adhesions.

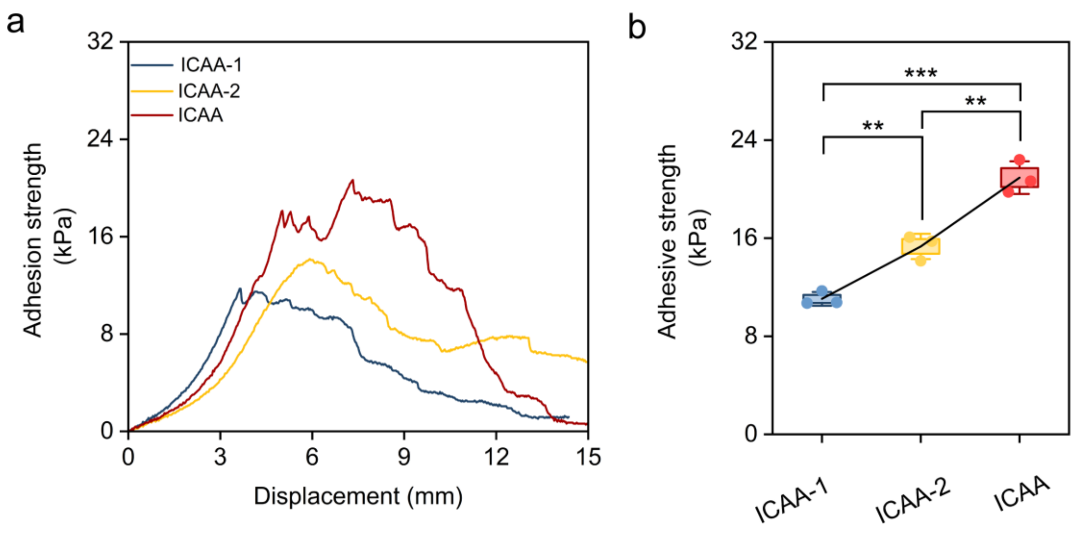


**Supplementary Fig. 11 Adhesion of the ICAA hydrogel with sciatic nerve tissues.** **a** Representative stress-displacement curves of ICAA hydrogel-tissue interface in lap-shear measurements. **b** Adhesion strength of ICAA hydrogel with nerve tissue (n) (n = 3 independent experiments), show significantly improvement with the increase in its solid content. Data are presented as the mean ± standard deviation in (**b**) and were analyzed by one-way ANOVA first, and then by the Tukey′s post hoc test. $^{**}P \leq 0.01$, $^{***}P \leq 0.001$. **b** $p = 5.31 \times 10^{-3}$ (ICAA-2 vs ICAA-1), $p = 5.54 \times 10^{-5}$ (ICAA vs ICAA-1), $p = 1.32 \times 10^{-3}$ (ICAA vs ICAA-2).

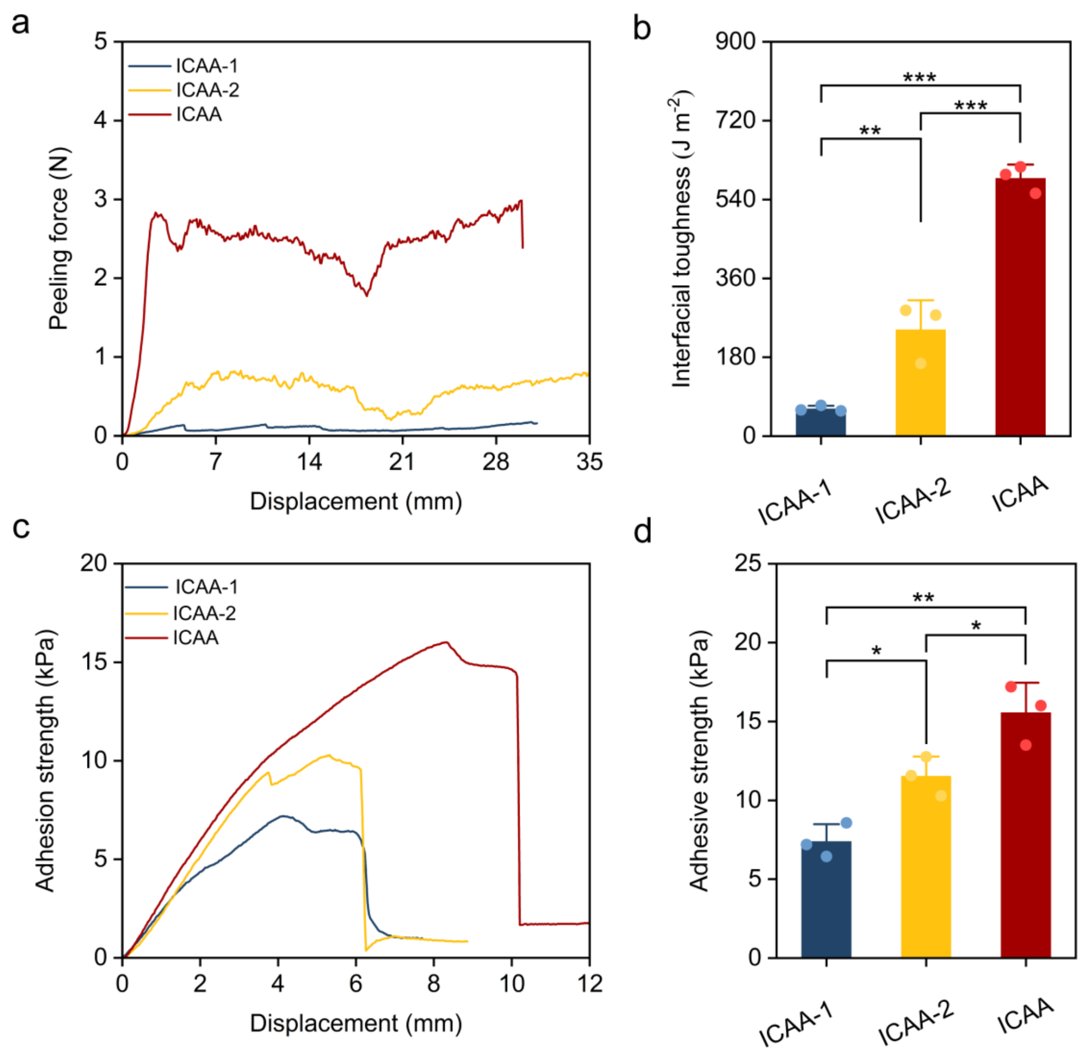


**Supplementary Fig. 12 Adhesion of the ICAA hydrogel with PDA-coated substrates. a, c** Representative force-displacement curves for hydrogel-tissue hybrids in peeling (a) and lap-shear tests (c). **b, d** Interfacial toughness (b) and adhesion strength (d) between ICAA hydrogel and nerve tissue (n = 3 independent animals). The interfacial toughness and adhesion strength of the ICAA hydrogel with PDA-coated substrates significantly improved with an increase in its solid content. Data are presented as the mean ± standard deviation in (**b**, **d**) and were analyzed by one-way ANOVA first, and then by the Tukey′s post hoc test. *P ≤ 0.05, **P ≤ 0.01, ***P ≤ 0.001. **b** $p = 5.09 \times 10^{-3}$ (ICAA-2 vs ICAA-1), $p = 1.36 \times 10^{-5}$ (ICAA vs ICAA-1), $p = 1.54 \times 10^{-4}$ (ICAA vs ICAA-2). **d** $p = 2.93 \times 10^{-2}$ (ICAA-2 vs ICAA-1), $p = 1.09 \times 10^{-3}$ (ICAA vs ICAA-1), $p = 3.29 \times 10^{-2}$ (ICAA vs ICAA-2).

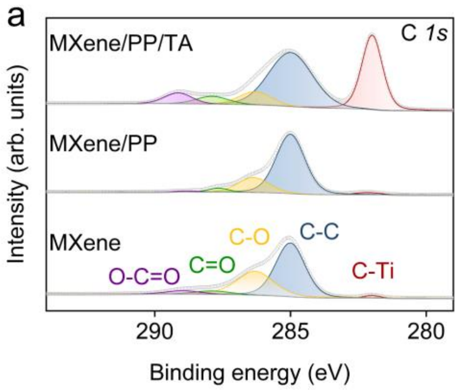


b

| | | Mxene | | Mxene/PP | | Mxene/PP/TA | |
|---|---|---|---|---|---|---|---|
| Core level | Assignment | Position (eV) | Percentage | Position (eV) | Percentage | Position (eV) | Percentage |
| C *1s* | C-C | 285 | 56.3% | 285 | 73% | 285 | 65.4% |
| | C-Ti | 282 | 1.5% | 282.1 | 2.5% | 282.2 | 24% |
| | C-O | 286.3 | 33.4% | 286.4 | 19.9% | 286.3 | 4% |
| | C=O | 287.9 | 4.5% | 287.7 | 3.5% | 287.9 | 1.7% |
| | O-C=O | 288.9 | 4.3% | 288.8 | 1.1% | 289.2 | 4.9% |

**Supplementary Fig. 13 XPS analysis of MXene, MXene/PP and MXene/PP/TA. a** C *1s* XPS spectra of MXene, MXene/PP and MXene/PP/TA. Binding energies were all calibrated to the C *1s* peak at 285 eV. **b** Assignments of C *1s* XPS spectra of Mxene, Mxene/PP and Mxene/PP/TA.

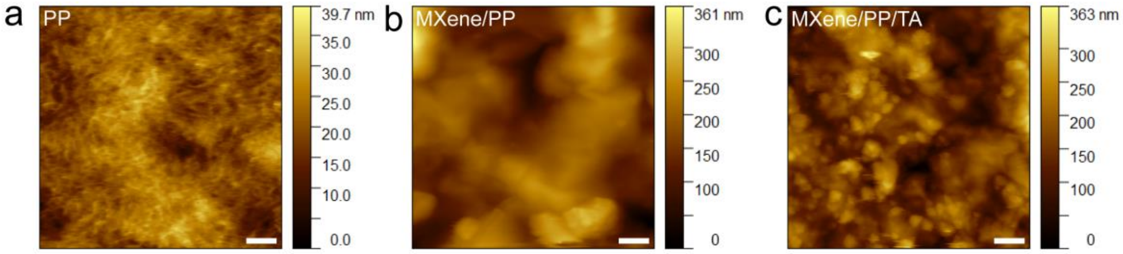


**Supplementary Fig. 14 AFM analysis of PEDOT:PSS (PP), MXene/PP and MXene/PP/TA.** **a** Height image of PP. **b** Height image of Mxene/PP. **c** Height image of Mxene/PP/TA. Scale bar, 250 nm.

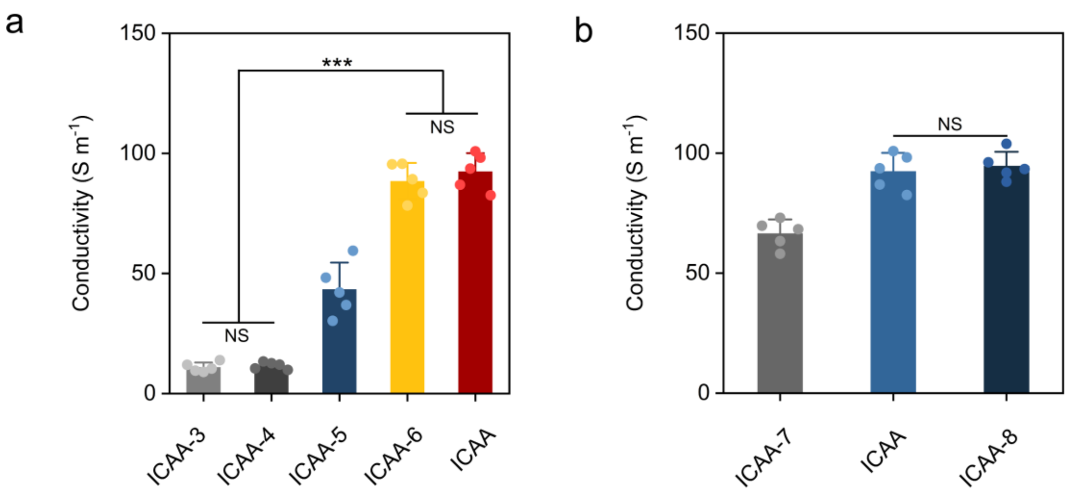


**Supplementary Fig. 15 Conductivities of the ICAA hydrogel with different components. a** Influence of MXene content on the conductivity of ICAA hydrogel (n = 5 independent experiments). **b** Influence of TA content on the conductivity of ICAA hydrogel (n = 5 independent experiments). Data are presented as the mean ± standard deviation in (**a, b**) and were analyzed by one-way ANOVA first, and then by the Tukey′s post hoc test. ***P ≤ 0.001. NS, not significant. **a** $p$ = 0.9998 (ICAA-4 vs ICAA-3), $p = 9.28 \times 10^{-11}$ (ICAA-6 vs ICAA-3), $p = 3.06 \times 10^{-10}$ (ICAA-6 vs ICAA-4). $p$ = 0 (ICAA vs ICAA-3), $p$ = 0 (ICAA vs ICAA-4), $p$ = 0.8933 (ICAA vs ICAA-6). **b** $p = 1.12 \times 10^{-4}$ (ICAA vs ICAA-7), $p = 5.10 \times 10^{-5}$ (ICAA-8 vs ICAA-7), $p$ = 0.853 (ICAA-8 vs ICAA).

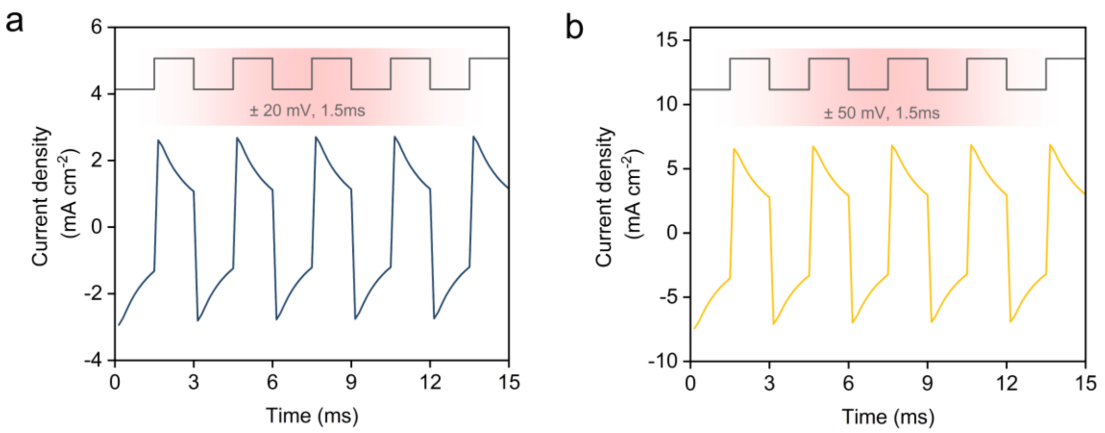


**Supplementary Fig. 16 CIC curves of the ICAA hydrogel with low stimulation voltage. a** High current density of ~2.5 mA $cm^{-2}$ was achieved with a stimulation voltage as low as ± 20 mV. **b** high current density of ~6.5 mA $cm^{-2}$ was achieved with a stimulation voltage as low as ± 50 mV.

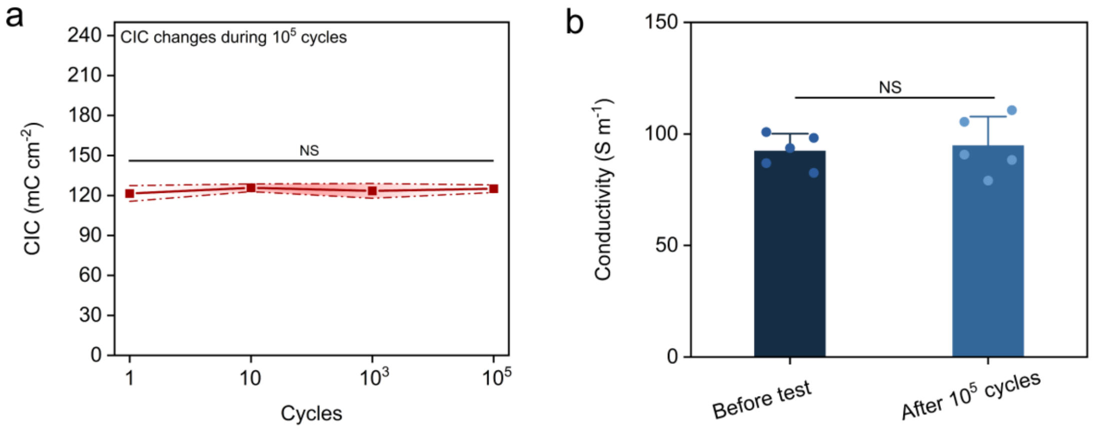


**Supplementary Fig. 17 Electrochemical properties of the ICAA hydrogel under prolonged electrical stimulation.** a Changes of the ICAA hydrogel′s CIC during $10^5$ charging and discharging cycles indicate its long-term stability under prolonged electrical stimulation (n = 3 independent experiments). b Conductivities of the ICAA hydrogel before and after $10^5$ charging and discharging cycles show insignificant changes (n = 5 independent experiments). Data are presented as the mean ± standard deviation in (**a, b**) and were analyzed by one-way ANOVA first, and then by the Tukey′s post hoc test. ***P ≤ 0.001. NS, not significant. **a** $p = 0.6652$ (Cycle 10 vs Cycle 1), $p = 0.9462$ (Cycle $10^3$ vs Cycle 1), $p = 0.9229$ (Cycle $10^3$ vs Cycle 10). $p = 0.7687$ (Cycle $10^5$ vs Cycle 1), $p = 0.9974$ (Cycle $10^5$ vs Cycle 10), $p = 0.9716$ (Cycle $10^5$ vs Cycle $10^3$). **b** $p = 0.7292$ (Before test vs After $10^5$ cycles).

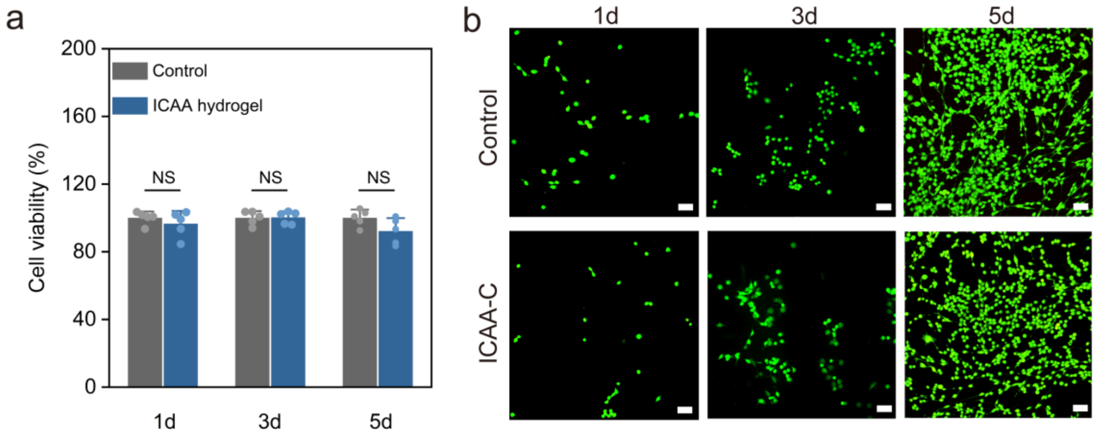


**Supplementary Fig. 18 Cytocompatibility of the ICAA hydrogel. a** CCK8-detected cell viability of PC12 cells with the ICAA extracts (n = 5 independent experiments). **b** Live-dead staining of PC12 cells with the ICAA extracts. Scale bars, 100 μm. Data are presented as the mean ± standard deviation and were analyzed using one-way ANOVA with Tukey′s post hoc test in (**a**). NS, not significant. **a** $p$ = 0.3879 (ICAA hydrogel (1d) vs Control (1d)), $p$ = 0.9229 (ICAA hydrogel (2d) vs Control (2d)), $p$ = 0.0936 (ICAA hydrogel (3d) vs Control (3d)).

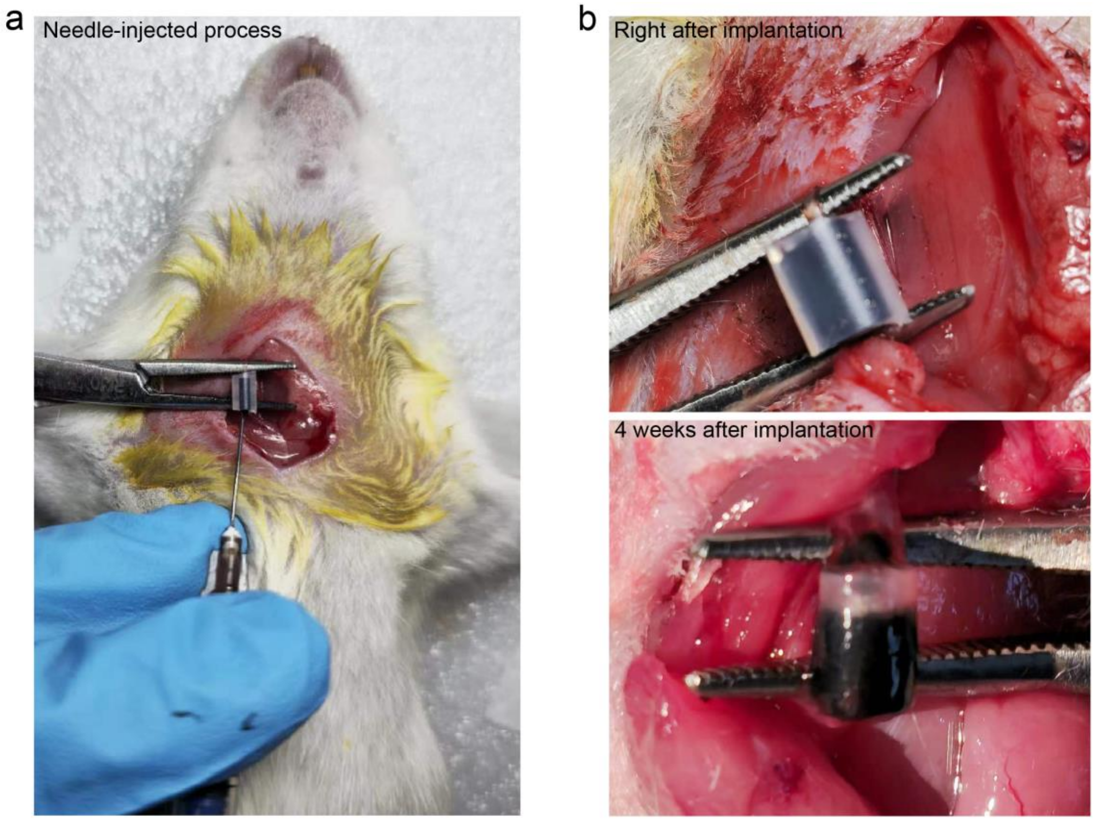


**Supplementary Fig. 19 Injectable process of the ICAA hydrogel. a** Image of injection procedure of the ICAA hydrogel show it can be simply injected by syringe needle. **b** Shapes of the ICAA hydrogel right after implantation and 4 weeks after implantation show its *in vivo* shape stability in a dynamic biological environment.

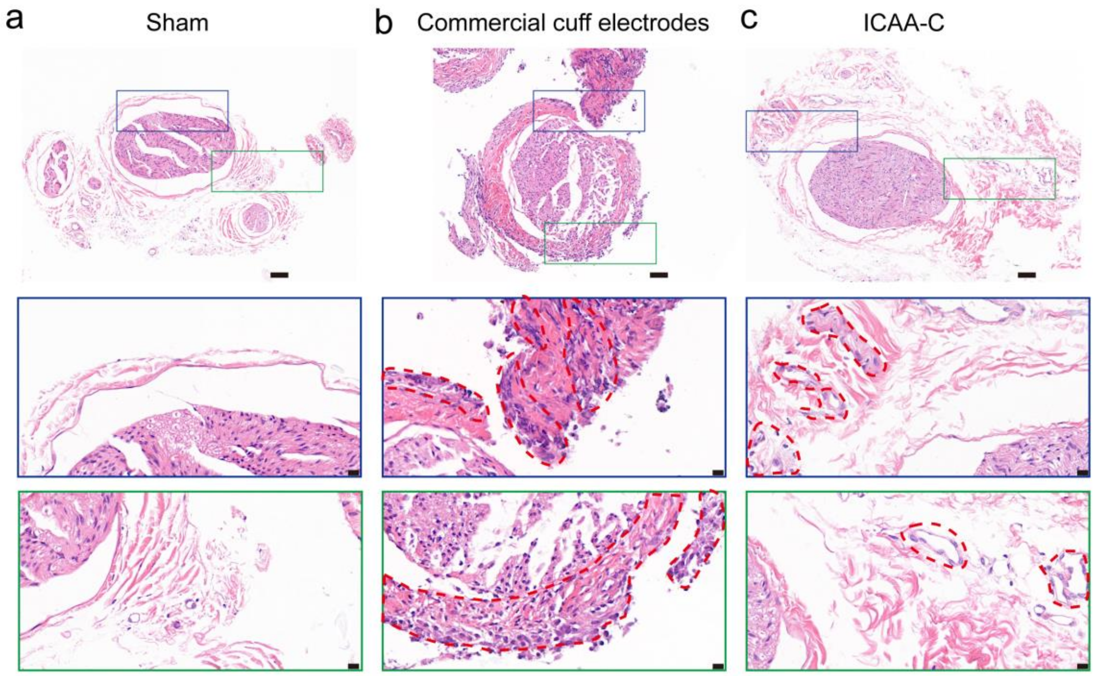


**Supplementary Fig. 20 Histological analysis of vagus nerves at the device-implanted sites.** H&E-stained histological appearance of the vagus nerves dissected in sham group (a), commercial cuff electrodes group (b), and ICAA-C group (c). The image was magnified ×20 (top), ×80 (middle and bottom) to show upper (blue) and lower (green) regions of the vagus nerve. The fibrosis regions are marked with red dotted boxes. Scale bars, 50 μm (top), 10 μm (middle and bottom).

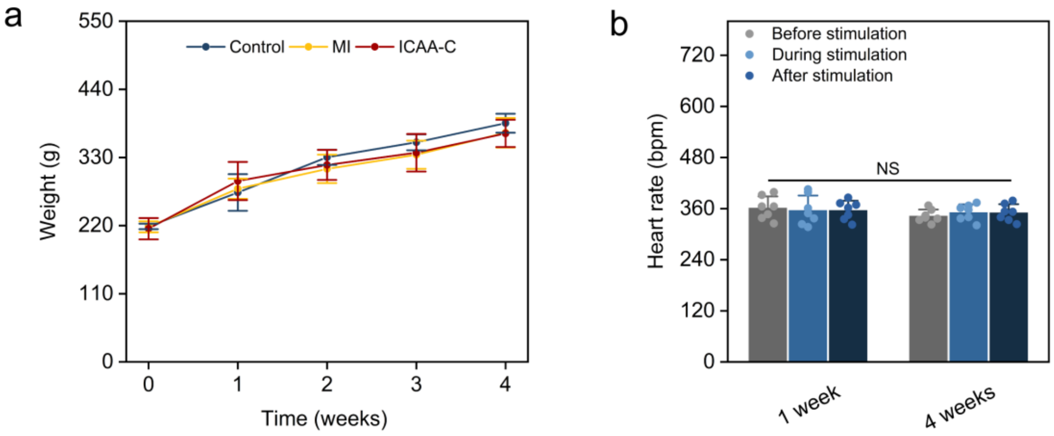


**Supplementary Fig. 21 Monitoring of health status of rats during vagus nerve stimulation therapy. a** Weight change of rats in control, MI, ICAAC group during 4-week therapy (n = 7 independent animals). **b** Herat rate of rats in ICAA-C group before, during, and after vagus nerve stimulation during 4-week therapy (n = 7 independent animals). Data are presented as the mean ± standard deviation in (**a, b**) and were analyzed by one-way ANOVA first, and then by the Tukey′s post hoc test in (**b**). NS, not significant. **b** 1 week: $p$ = 0.92753 (During stimulation vs Before stimulation), $p$ = 0.9307 (After stimulation vs Before stimulation), $p$ = 0.99996 (After stimulation vs During stimulation). 4 weeks: $p$ = 0.67853 (During stimulation vs Before stimulation), $p$ = 0.7186 (After stimulation vs Before stimulation), $p$ = 0.99756 (After stimulation vs During stimulation).

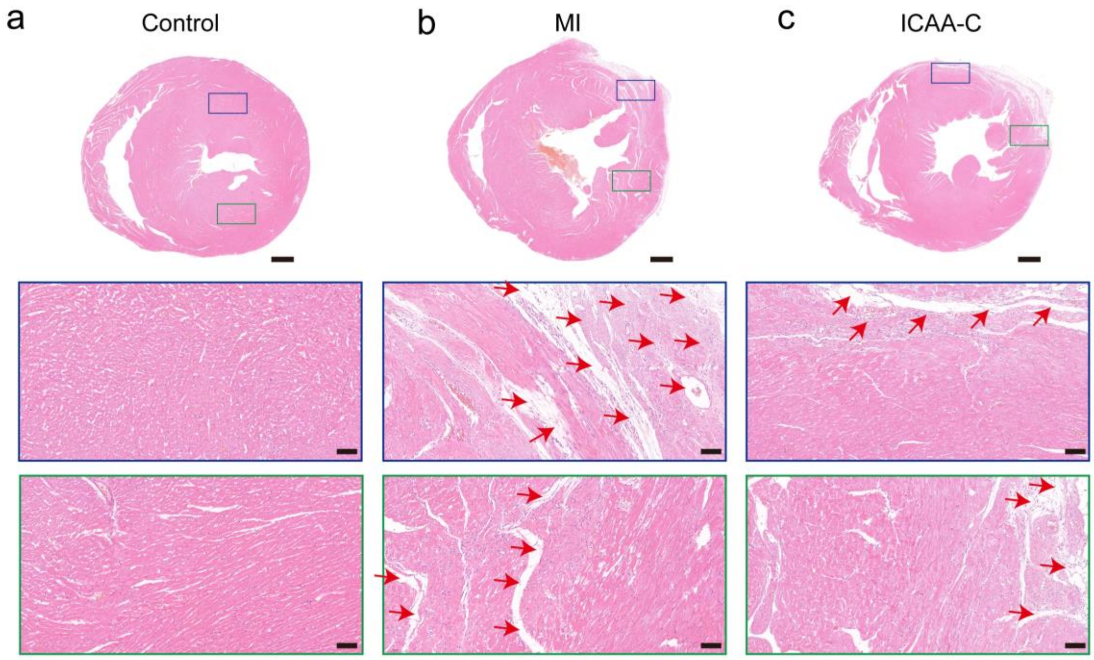


**Supplementary Fig. 22 Histological analysis of hearts.** H&E-stained histological appearance of the heart tissues dissected in control group (a), MI group (b), and ICAA-C group (c). The image was magnified ×1 (top), ×15 (middle and bottom) to show upper (blue) and lower (green) regions of the left ventricular wall. Scale bars, 1 mm (top), 100 μm (middle and bottom). H&E staining revealed mild inflammation and immune responses (pointed out by red arrows), characterized by myocardial cell necrosis, loose tissue and increased transparency.

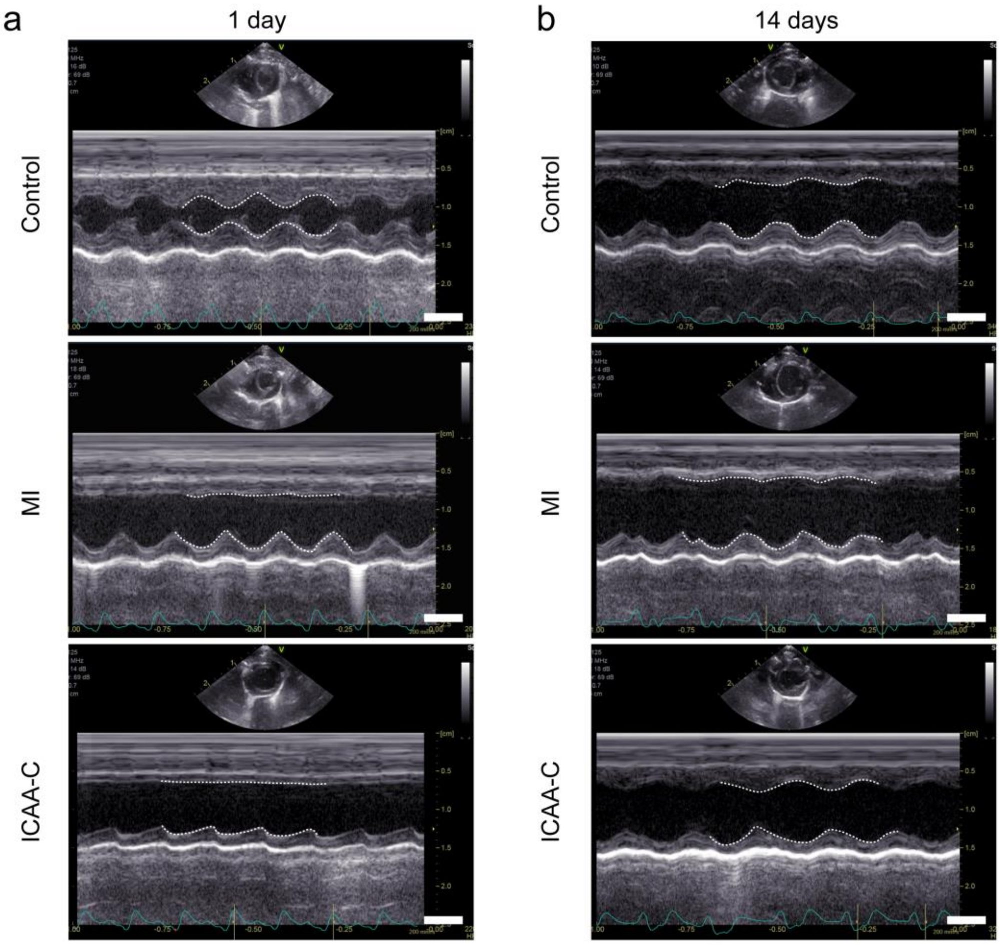


**Supplementary Fig. 23 Echocardiography imaging of rats in the control, MI, and ICAA-C groups at day 1 (a) and day 14 (b) after modeling.** The dotted lines showed the size changes of the left ventricle in the cross-section at the level of the papillary muscles during systole and diastole. Scale bar, 5 mm.

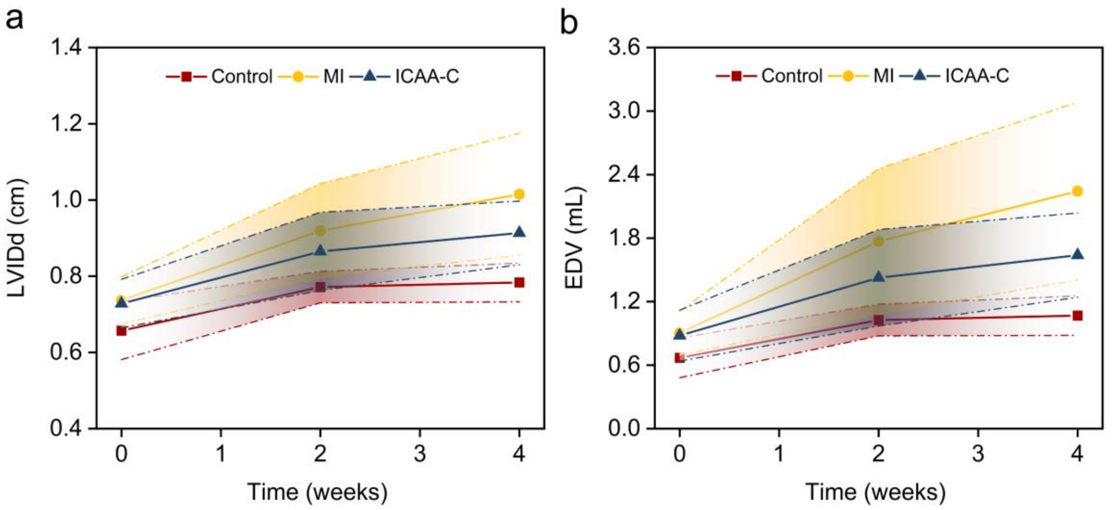


**Supplementary Fig. 24 End-diastolic diameters (a) and volumes (b) of hearts in the ICAA-C, MI, and control groups within 4 weeks.** Data are presented as the mean ± standard deviation in (**a**, **b**) (n=7 independent animals).

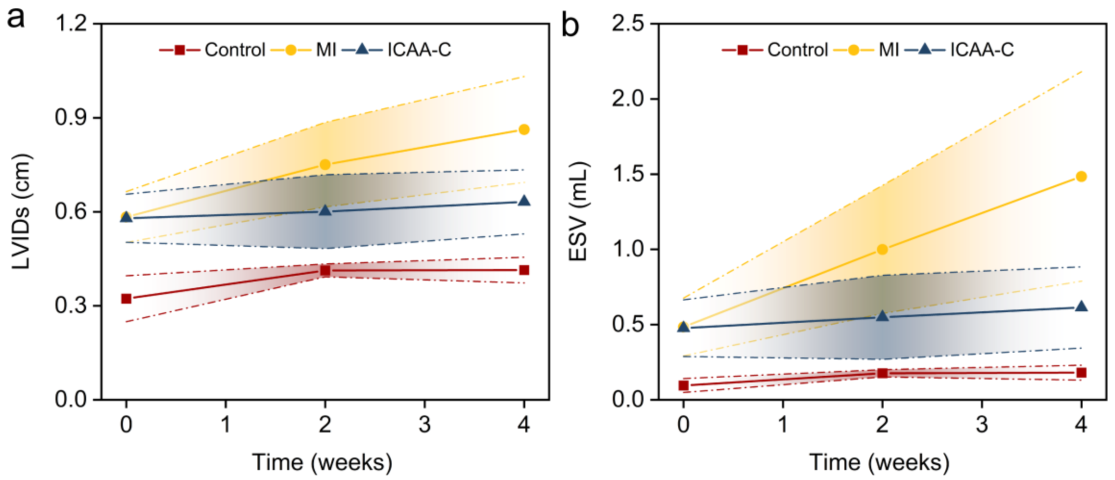


**Supplementary Fig. 25 End-systolic diameters (a) and volumes (b) of hearts in the ICAA-C, MI, and control groups within 4 weeks.** Data are presented as the mean ± standard deviation in (**a**, **b**) (n = 7 independent animals).

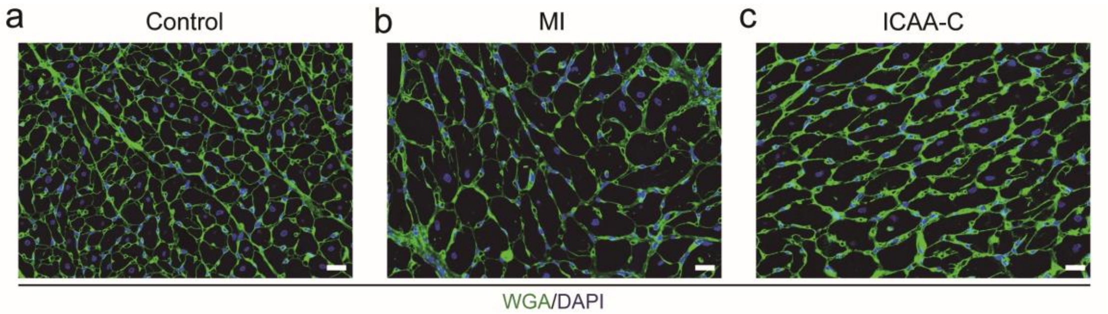


**Supplementary Fig. 26 Representative immunofluorescence images of the infarct area in the ICAA-C, MI, and control groups for wheat germ agglutinin (WGA).** ICAA-C-based vagus nerve stimulation suppress their enlargement of cardiomyocytes after MI. Scale bar, 20 μm.

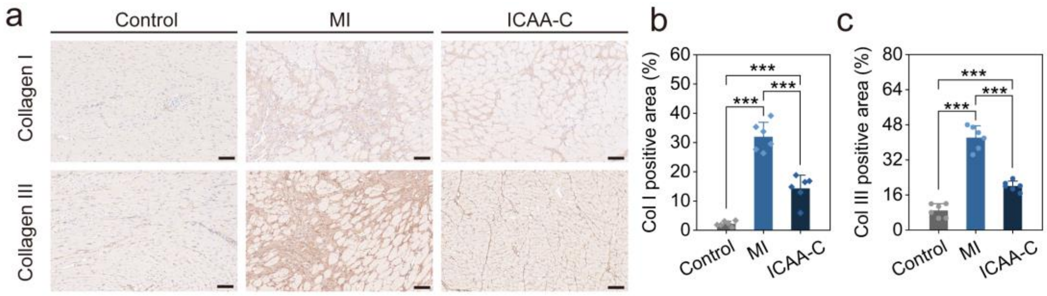


**Supplementary Fig. 27 Immunohistochemical analysis of collagen expression in the ICAA-C, MI, and control groups. a** Representative immunohistochemical images of Collagen I (Col I) and Collagen III (Col III) in the ICAA-C, MI, and control groups. Scale bar, 50 μm. **b, c** Quantitative analysis of Col I positive area% (b) (n=6 independent animals), Col III positive area% (c) (n=6 independent animals). Data are presented as the mean ± standard deviation in (**b**, **c**) and were analyzed using one-way ANOVA with Tukey′s post hoc test. ***P ≤ 0.001. **b** $p = 0$ (MI vs Control), $p = 2.25 \times 10^{-4}$ (ICAA-C vs Control), $p = 3.15 \times 10^{-6}$ (ICAA -C vs MI). **c** $p = 0$ (MI vs Control), $p = 4.03 \times 10^{-4}$ (ICAA-C vs Control), $p = 8.35\times 10^{-8}$ (ICAA -C vs MI).

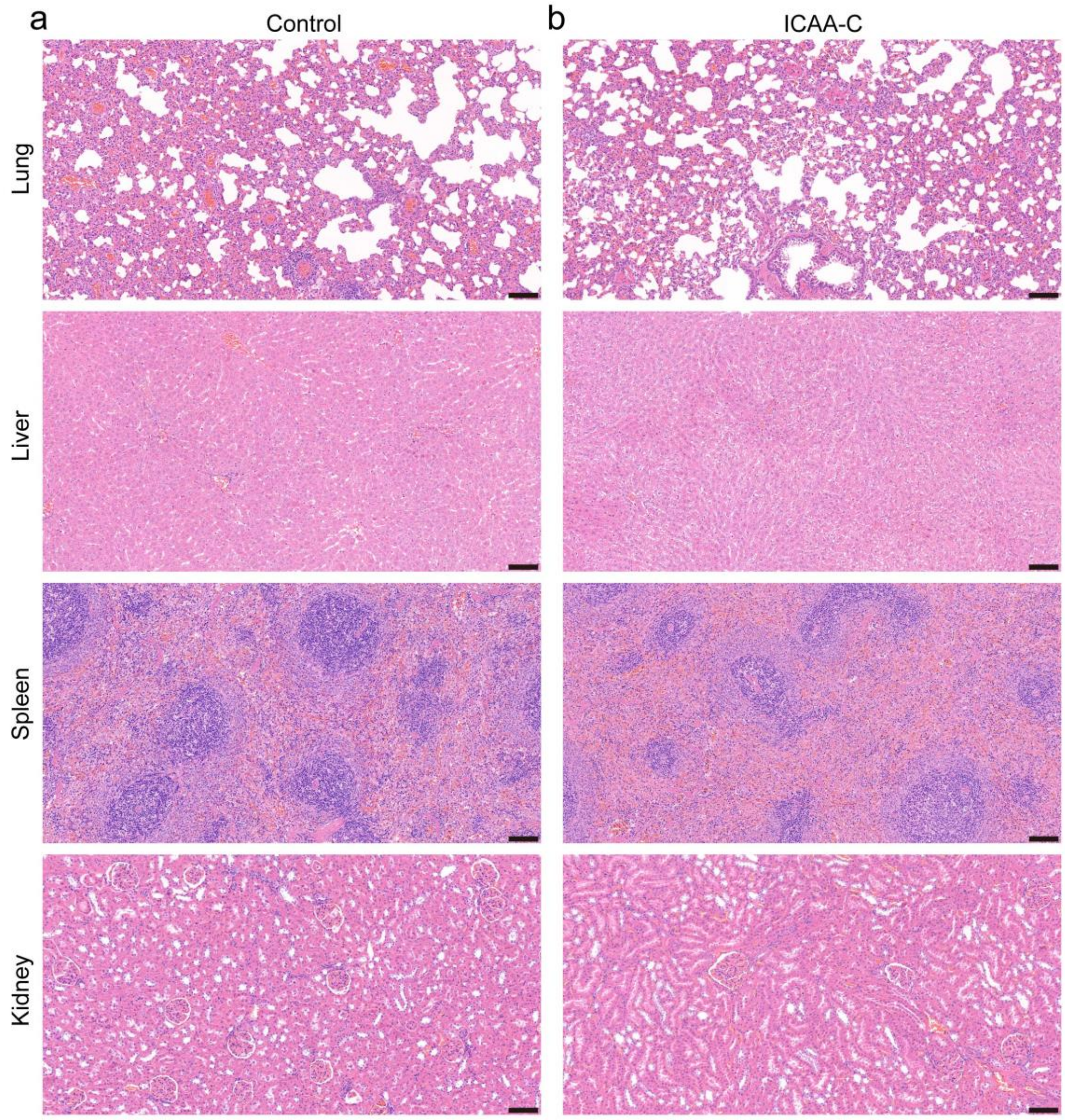


**Supplementary Fig. 28 Histological analysis of different organs.** H&E-stained histological appearance of the lung, liver, spleen, kidney tissues dissected in control group (a) and ICAA-C group (b). scale bar, 100 μm.

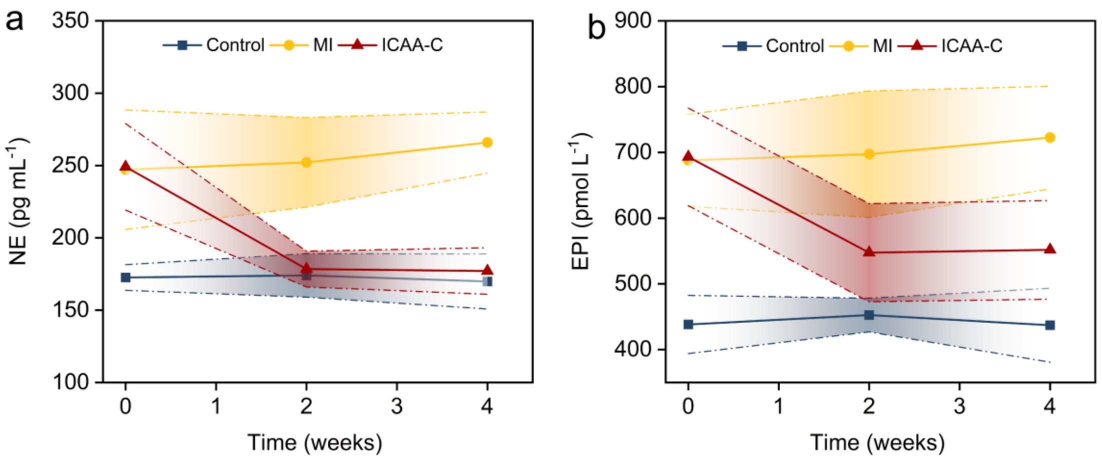


**Supplementary Fig. 29 Effects of ICAA-C-enabled vagus nerve stimulation on neurotransmitters released by the sympathetic nerve.** (a) Serum level of norepinephrine (NE) at 1 day, 2 weeks, and 4 weeks post-operation in the ICAA-C, MI, and control groups (n = 7 independent animals). (b) Serum level of epinephrine (EPI) at 1 day, 2 weeks, and 4 weeks post-operation in the ICAA-C, MI, and control groups (n = 7 independent animals). Data are presented as the mean ± standard deviation in (**a, b**).

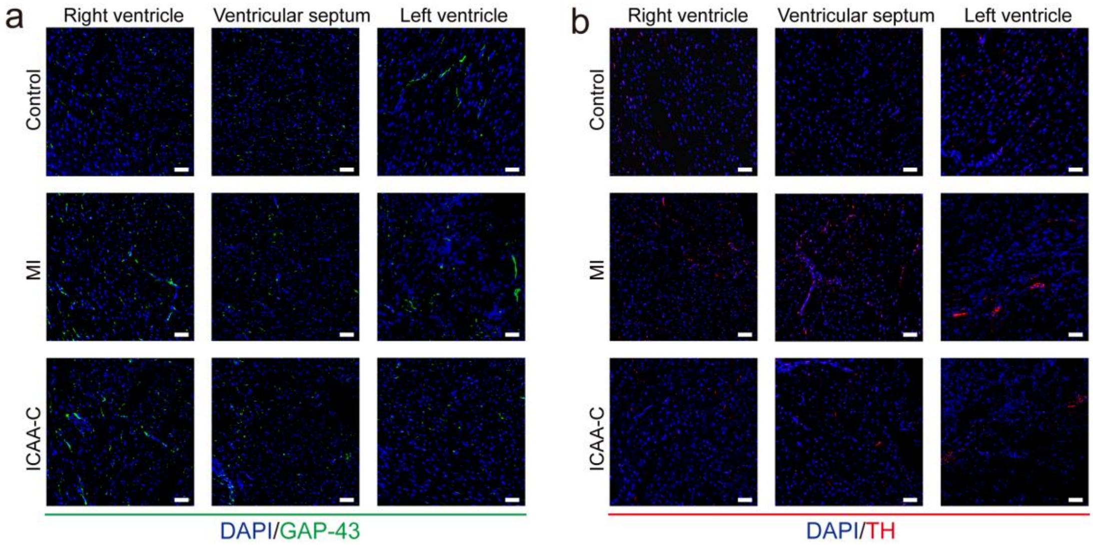


**Supplementary Fig. 30 Representative immunofluorescence images of GAP-43 and TH. a** Representative immunofluorescence staining of the left ventricle, right ventricle, and ventricular septum of rat heart in the ICAA-C, MI, and control groups for growth-associated protein 43 (GAP-43). Scale bar, 50 μm. **b** Representative immunofluorescence staining of the left ventricle, right ventricle, and ventricular septum of rat heart in the ICAA-C, MI, and control groups for tyrosine hydroxylase (TH). Scale bar, 50 μm.

**Supplementary Table 1** Component content of the ICAA hydrogels with different solid contents.

| Hydrogel | 8 -arm PEG-SH（wt%） | PEG-2mal (wt%) | MXene (wt%) | PEDOT:PSS (wt%) | TA (wt%) |
|---|---|---|---|---|---|
| ICAA | 15 | 10 | 4 | 1.1-1.3 | 0.3 |
| ICAA-2 | 10 | 6.66 | 2.66 | 1.1-1.3 | 0.3 |
| ICAA-1 | 5 | 3.33 | 1.33 | 1.1-1.3 | 0.3 |

**Supplementary Table 2** Assignments of Ti *2p* XPS spectra of MXene, MXene/PP and MXene/PP/TA.

| | | MXene | | MXene/PP | | MXene/PP/TA | |
|---|---|---|---|---|---|---|---|
| Core level | Assignment | Position (eV) | Percentage | Position (eV) | Percentage | Position (eV) | Percentage |
| Ti $2p_{3/2}$ | Ti-C | 455 | 23.3% | 455.4 | 27.8% | 455.5 | 30.6% |
| Ti $2p_{1/2}$ | Ti-C | 460.7 | | 461.1 | | 461.2 | |
| Ti $2p_{3/2}$ | Ti (II) | 455.9 | 34.2% | 456.4 | 35.9% | 456.4 | 32% |
| Ti $2p_{1/2}$ | Ti (II) | 461.6 | | 462.1 | | 462.1 | |
| Ti $2p_{3/2}$ | Ti (III) | 457.6 | 9.2% | 458 | 18.1% | 458.1 | 23% |
| Ti $2p_{1/2}$ | Ti (III) | 463.4 | | 463.7 | | 463.8 | |
| Ti $2p_{3/2}$ | Ti (IV) | 459.3 | 31.4% | 459.6 | 15.4% | 459.6 | 11.8% |
| Ti $2p_{1/2}$ | Ti (IV) | 465.1 | | 465.3 | | 465.3 | |
| Ti $2p_{3/2}$ | C-Ti-Fx | 460.6 | 1.9% | 460.9 | 2.8% | 461 | 2.6% |
| Ti $2p_{1/2}$ | C-Ti-Fx | 466.3 | | 466.6 | | 466.7 | |

**Supplementary Table 3** Component content of the ICAA hydrogels with different conductive components and TA contents.

| Hydrogel | 8-arm PEG-SH（wt%） | PEG-2mal (wt%) | MXene (wt%) | PEDOT：PSS (wt%) | TA (wt%) |
|---|---|---|---|---|---|
| ICAA | 15 | 10 | 4 | 1.1-1.3 | 0.3 |
| ICAA-3 | 15 | 10 | 0.5 | 1.1-1.3 | 0.3 |
| ICAA-4 | 15 | 10 | 1 | 1.1-1.3 | 0.3 |
| ICAA-5 | 15 | 10 | 2 | 1.1-1.3 | 0.3 |
| ICAA-6 | 15 | 10 | 3 | 1.1-1.3 | 0.3 |
| ICAA-7 | 15 | 10 | 3 | 1.1-1.3 | 0.1 |
| ICAA-8 | 15 | 10 | 3 | 1.1-1.3 | 0.5 |

**Supplementary Table 4** Comparison of the ICAA hydrogel with previously reported injectable, conductive hydrogels.

| Hydrogel | Injectability | Mechanical properties | Conductivity | Wet tissue adhesion | Anti-swelling | Stability | Reference |
|---|---|---|---|---|---|---|---|
| ICAA | Irreversible covalent bonds and reversible noncovalent bonds | 6.42-40.9 kPa | 92.43 ± 7.65 S $m^{-1}$ | 20.9 kPa | √ | Mechanical (√) Electrical (√) | This work |
| CAHPs | Reversible covalent bonds and reversible noncovalent bonds | 10-40 kPa | 1.35 ± 0.32 S $m^{-1}$ | 4.84-13.65 kPa | √ | Mechanical (/) Electrical (√) | 1 |
| IT-IC | Irreversible covalent bonds and reversible noncovalent bonds | 0.1889 ± 0.0283 kPa | 10 S $m^{-1}$ | × | × | Mechanical (×) Electrical (×) | 2 |
| HPAE-Py (50%)/Geln | Reversible covalent bonds and reversible noncovalent bonds | 34.7 kPa | 0.065 ± 0.0012 S $m^{-1}$ | 22.2 kPa | × | Mechanical (×) Electrical (×) | 3 |
| EGC20 | Irreversible covalent bonds | 3-45 kPa | / | × | × | Mechanical (×) Electrical (×) | 4 |
| γ-PGA/PEDOT: PSS | Reversible noncovalent bonds | 383 kPa | 12.5 S $m^{-1}$ | / | × | Mechanical (×) Electrical (×) | 5 |
| RT-PEDOT: PSS | Reversible noncovalent bonds | 1 kPa | 10 S $m^{-1}$ | × | × | Mechanical (×) Electrical (×) | 6 |

## Supplementary References


1. Yu C, *et al.* Chronological adhesive cardiac patch for synchronous mechanophysiological monitoring and electrocoupling therapy. *Nat. Commun.* **14**, 6226 (2023).
2. Jin S, *et al.* Injectable tissue prosthesis for instantaneous closed-loop rehabilitation. *Nature* **623**, 58-65 (2023).
3. Liang S, *et al.* Paintable and Rapidly Bondable Conductive Hydrogels as Therapeutic Cardiac Patches. *Adv. Mater.* **30**, e1704235 (2018).
4. Wang L, *et al.* Injectable and conductive cardiac patches repair infarcted myocardium in rats and minipigs. *Nat. Biomed. Eng.* **5**, 1157-1173 (2021).
5. Zhang C, *et al.* Highly adhesive and self-healing γ-PGA/PEDOT:PSS conductive hydrogels enabled by multiple hydrogen bonding for wearable electronics. *Nano Energy* **95**, 106991 (2022).
6. Zhang S, *et al.* Room-Temperature-Formed PEDOT:PSS Hydrogels Enable Injectable, Soft, and Healable Organic Bioelectronics. *Adv. Mater.* **32**, e1904752 (2020).
