# Supplementary material for: Highly-stable, injectable, conductive hydrogel for chronic neuromodulation
Source: Nat Commun. 2024 Sep 12;15:7993. doi: 10.1038/s41467-024-52418-y (PMC11393409; doi:10.1038/s41467-024-52418-y)
Supplement: Supplementary file 2 — Reporting Summary [file 41467_2024_52418_MOESM2_ESM.pdf]

nature portfolio

Corresponding author(s): Li Zhang, Yuanwen Jiang, Zhiqiang Luo

Last updated by author(s): Aug 17, 2024

Reporting Summary

Nature Portfolio wishes to improve the reproducibility of the work that we publish. This form provides structure for consistency and transparency in reporting. For further information on Nature Portfolio policies, see our Editorial Policies and the Editorial Policy Checklist.

Statistics

For all statistical analyses, confirm that the following items are present in the figure legend, table legend, main text, or Methods section.

n/a	Confirmed	
☐	☒	The exact sample size (*n*) for each experimental group/condition, given as a discrete number and unit of measurement
☐	☒	A statement on whether measurements were taken from distinct samples or whether the same sample was measured repeatedly
☐	☒	The statistical test(s) used AND whether they are one- or two-sided<br>*Only common tests should be described solely by name; describe more complex techniques in the Methods section.*
☒	☐	A description of all covariates tested
☒	☐	A description of any assumptions or corrections, such as tests of normality and adjustment for multiple comparisons
☐	☒	A full description of the statistical parameters including central tendency (e.g. means) or other basic estimates (e.g. regression coefficient) AND variation (e.g. standard deviation) or associated estimates of uncertainty (e.g. confidence intervals)
☐	☒	For null hypothesis testing, the test statistic (e.g. *F*, *t*, *r*) with confidence intervals, effect sizes, degrees of freedom and *P* value noted<br>*Give P values as exact values whenever suitable.*
☒	☐	For Bayesian analysis, information on the choice of priors and Markov chain Monte Carlo settings
☒	☐	For hierarchical and complex designs, identification of the appropriate level for tests and full reporting of outcomes
☒	☐	Estimates of effect sizes (e.g. Cohen's *d*, Pearson's *r*), indicating how they were calculated

Our web collection on statistics for biologists contains articles on many of the points above.

Software and code

Policy information about availability of computer code

Data collection	Nuclear magnetic resonance spectrometer (AV400, Bruker, Switzerland), Laser confocal Raman spectrometer (LabRAM HR800, Horiba Jobin Yvon, France), X-ray photoelectron spectroscopy (AXIS-ULTRA DLD, Kratos, Japan), Atomic force microscopic (Jupiter XR, Asylum Research, US), Rheometer (MCR102, Anton Paar, Austria), Universal testing machine (CTM8000, Xie Qiang Instrument Manufacturing), Four-point probe instrument (KDY-1, Kunde Semiconductor Co., Ltd), Electrochemical workstation (CHI660E, Shanghai Chenhua Apparatus Shanghai Chenhua Co., Ltd), Biological experiment system (BL420N, Chengdu Techman), Multi-function microplate reader (PARK 10M, TECAN, Switzerland)
Data analysis	Origin 2023 software were used for data plotting and statistical analysis. Image J software was used for quantitative imaging analyses. XPSpeak 41 was used for XPS analysis.

For manuscripts utilizing custom algorithms or software that are central to the research but not yet described in published literature, software must be made available to editors and reviewers. We strongly encourage code deposition in a community repository (e.g. GitHub). See the Nature Portfolio guidelines for submitting code & software for further information.

Data

Policy information about availability of data

All manuscripts must include a data availability statement. This statement should provide the following information, where applicable:

- Accession codes, unique identifiers, or web links for publicly available datasets
- A description of any restrictions on data availability
- For clinical datasets or third party data, please ensure that the statement adheres to our policy

All data supporting the findings of this study are available within the paper and its supplementary information or from the corresponding authors on request. Source data are provided with this paper.

Research involving human participants, their data, or biological material

Policy information about studies with human participants or human data. See also policy information about sex, gender (identity/presentation), and sexual orientation and race, ethnicity and racism.

Reporting on sex and gender	This work did not involve human research.
Reporting on race, ethnicity, or other socially relevant groupings	This work did not involve human research.
Population characteristics	This work did not involve human research.
Recruitment	This work did not involve human research.
Ethics oversight	This work did not involve human research.

Note that full information on the approval of the study protocol must also be provided in the manuscript.

Field-specific reporting

Please select the one below that is the best fit for your research. If you are not sure, read the appropriate sections before making your selection.

- [x] Life sciences
- [ ] Behavioural & social sciences
- [ ] Ecological, evolutionary & environmental sciences

For a reference copy of the document with all sections, see nature.com/documents/nr-reporting-summary-flat.pdf

Life sciences study design

All studies must disclose on these points even when the disclosure is negative.

Sample size	Details regarding the sample size of all experiments are provided in the Methods section and figure legends. For material characterization, we determined the sample size based on previous experience and other publications . Each sample was tested at least three times to ensure that they were sufficient for statistical comparison between different groups. The sample was randomly chosen to be determined. The significance level was set as 0.05, and the sample size was calculated as 6 rats per group. Animal experiments were performed on 6 rats per group, and electrocardiography were performed on 7 rats in each group at different times.
Data exclusions	No data were excluded from this study.
Replication	All experiments were performed with independent replicates as described in the figure legends.
Randomization	All samples were randomly allocated into experimental groups.
Blinding	Investigators were blinded to group allocation during data collection and analysis.

Reporting for specific materials, systems and methods

We require information from authors about some types of materials, experimental systems and methods used in many studies. Here, indicate whether each material, system or method listed is relevant to your study. If you are not sure if a list item applies to your research, read the appropriate section before selecting a response.

Materials & experimental systems

n/a	Involved in the study	
	x	Antibodies
	x	Eukaryotic cell lines
x		Palaeontology and archaeology
	x	Animals and other organisms
x		Clinical data
x		Dual use research of concern
x		Plants

Methods

n/a	Involved in the study	
x		ChIP-seq
x		Flow cytometry
x		MRI-based neuroimaging

Antibodies

Antibodies used

Primary antibodies:
Anti-Iba1 Rabbit pAb (Iba-1; Recombinant protein corresponding to Mouse Iba1; Servicebio; Catalog No. GB113502);
Anti-160 kDa Neurofilament Medium Mouse mAb (NFM; Recombinant protein corresponding to Mouse NF-M; Catalog No. GB12763-100);
Anti-S100 beta Rabbit pAb (S100; KLH conjugated Synthetic peptide corresponding to Mouse S100B; Catalog No. GB11359-100);
Anti-Collagen I Rabbit pAb (Collagen I; Recombinant protein corresponding to Mouse COL1A1; Catalog No. GB11022-3-100);
Anti-Collagen III Rabbit pAb (Collagen III; Recombinant protein corresponding to Mouse Collagen III; Catalog No. GB111629-100);
Anti-MMP9 Rabbit pAb (MMP9; KLH conjugated Synthetic peptide corresponding to Mouse MMP9; Catalog No. GB11132-100);
Anti-TGF beta 1 Rabbit pAb (TGF-β; KLH conjugated Synthetic peptide corresponding to Mouse TGF beta 1; Catalog No. GB11179-100);
Anti-GAP43 Rabbit pAb (GAP43; KLH conjugated Synthetic peptide corresponding to Mouse GAP43; Catalog No. GB11095-100);
Anti-Neurofilament heavy polypeptide Mouse mAb (NF200; KLH conjugated Synthetic peptide corresponding to Mouse NF200; Catalog No. GB12144-100)
Anti-Tyrosine Hydroxylase Rabbit pAb (TH; Recombinant protein corresponding to Mouse Tyrosine Hydroxylase; Catalog No. GB11181-100);
Anti-Connexin 43/GJA1 Mouse mAb (Cx43; KLH conjugated Synthetic peptide corresponding to Mouse Connexin 43; Catalog No. GB12234-100);
Anti-alpha Actinin Rabbit pAb (α-actin; KLH conjugated Synthetic peptide corresponding to Human ACTN1; Catalog No. GB111556-100);
Secondary antibodies:
Cy3 conjugated Goat Anti-Rabbit IgG (H+L) (Excitation and emission wavelength: 550nm and 570nm; Servicebio; Catalog No. GB21303);
Cy3 conjugated Goat Anti-mouseIgG(H+L) (Excitation and emission wavelength: 550nm and 570nm; Servicebio; Catalog No. GB21301);
HRP conjugated Goat Anti-Mouse IgG (H+L) (Servicebio; Catalog No. GB23301);
Alexa Fluor 488-conjugated Goat Anti-Rabbit IgG (H+L) (Excitation and emission wavelength: 493nm and 519nm; Servicebio; Catalog No. GB25303);

Validation

Anti-Iba1 Rabbit pAb (GB113502, Servicebio, dilution 1:500): Species Reactivity: Human, Mouse, Rat, Application: IHC/IF;
Anti-160 kDa Neurofilament Medium Mouse mAb (GB12763-100, Servicebio, dilution 1:200): Species Reactivity: Mouse, Rat, Application: WB, IHC/IF;
Anti-S100 beta Rabbit pAb (GB11359-100, Servicebio, dilution 1:200): Species Reactivity: Mouse, Rat, Application: IHC/IF;
Anti -Collagen I Rabbit pAb (GB11022-3-100, Servicebio, dilution 1:500): Species Reactivity: Human, Mouse, Rat, Application: IHC/IF;
Anti -Collagen III Rabbit pAb (GB111629-100, Servicebio, dilution 1:4): Species Reactivity: Human, Mouse, Rat, Application: WB, IHC/IF;
Anti-MMP9 Rabbit pAb (GB11132-100, Servicebio, dilution 1:500): Species Reactivity: Human, Mouse, Rat, Application: WB, IHC/IF;
Anti-TGF beta 1 Rabbit pAb (GB11179-100, Servicebio, dilution 1:200): Species Reactivity: Mouse, Rat, Application: IHC/IF;
Anti-GAP43 Rabbit pAb (GB11095-100, Servicebio, dilution 1:300): Species Reactivity: Human, Mouse, Rat, Application: WB, IHC/IF;
Anti-Neurofilament heavy polypeptide Mouse mAb (GB12144-100, Servicebio, dilution 1:300): Species Reactivity: Mouse, Rat, Application: WB, IHC/IF;
Anti-Tyrosine Hydroxylase Rabbit pAb (GB11181-100, Servicebio, dilution 1:200): Species Reactivity: Human, Mouse, Rat, Application: WB, IHC/IF;
Anti-Connexin 43/GJA1 Mouse mAb (GB12234-100, Servicebio, dilution 1:300): Species Reactivity: Human, Mouse, Rat, Application: IHC/IF;
Anti-alpha Actinin Rabbit pAb (GB111556-100; Servicebio; 1:300): Species Reactivity: Human, Mouse, Rat, Application: WB,IHC/IF;
Cy3 conjugated Goat Anti-Rabbit IgG (H+L) (GB21303; Servicebio; 1:300): Species Reactivity: Mouse, Rat, Application: IF/FC;
Cy3 conjugated Goat Anti-mouseIgG(H+L) (GB21301; Servicebio; 1:300): Species Reactivity: Mouse, Rat, Application: IF/FC;
HRP conjugated Goat Anti-Mouse IgG (H+L) (GB23301; Servicebio; 1:200): Species Reactivity: Mouse, Rat, Application: Elisa, WB, IHC;
Alexa Fluor 488-conjugated Goat Anti-Rabbit IgG (H+L) (GB25303; Servicebio; 1:400);

Eukaryotic cell lines

Policy information about cell lines and Sex and Gender in Research

Cell line source(s)	PC12 cell lines (catalog number: BNCC100235) were purchased from Bena Culture Collection (Henan, China) and derived from rattus norvegicus. The cell line source did not take gender into account.
Authentication	PC12 cell lines were not authenticated by the investigators in this paper. They were purchased from the company.
Mycoplasma contamination	PC12 cell lines were not tested for mycoplasma contamination.
Commonly misidentified lines (See ICLAC register)	No commonly misidentified cell lines were used in the study.

Animals and other research organisms

Policy information about studies involving animals; ARRIVE guidelines recommended for reporting animal research, and Sex and Gender in Research

Laboratory animals	Male Sprague Dawley rats (SD rats, ~200 g, 7-week-old) were used for all our experiments.
Wild animals	This study did not involve wild animals.
Reporting on sex	This study did not apply to only one sex. The study design and methods did not take sex into consideration. All male animals were used in this study, and sex differences were not considered. Animal experiments were not considered for sex analysis because myocardial structure and functions in the MI model and inflammatory responses in subcutaneous encapsulation were not related to sex.
Field-collected samples	This study did not involve samples collected from the field.
Ethics oversight	All animal experiments were conducted in accordance with guidelines approved by the Institutional Animal Care and Use Committee of Huazhong University of Science and Technology (IACUC Number: 3663).

Note that full information on the approval of the study protocol must also be provided in the manuscript.

Plants

Seed stocks	This study did not involve seed stocks.
Novel plant genotypes	This study did not involve plant genotypes.
Authentication	This study did not involve seed stocks or plant genotypes.
